# Modeling of the KOH-Polarization cells for mitigating the induced AC voltage in the metallic pipelines

**DOI:** 10.1016/j.heliyon.2020.e03417

**Published:** 2020-03-06

**Authors:** Mostafa A. Al-Gabalawy, Mohamed A. Mostafa, Abdel Salam Hamza, Shimaa A. Hussien

**Affiliations:** a Electrical Power and Control Department, Pyramids Higher Institute for Engineering and Technology, 6th October, Cairo, Egypt; b Electrical Power and Machines Department, MUST University, 6th October, Cairo, Egypt; c Electrical Power and Machines Department, Benha University, Shoubra, Cairo, Egypt; d Electrical Power and Machines Department, Princess Nourah Bint Abdulrahman University, Saudi Arabia

**Keywords:** AC corrosion, Induced AC voltage, Polarization cell, Cathodic protection, Pipeline

## Abstract

The problem of AC corrosion remains motivating for researchers because many factors influence the corrosion rate for buried pipelines due to the interference with overhead high voltage transmission lines (OHVTLs). Many researchers study the mechanisms of induced alternating current (AC) voltages, which are summarized as capacitive, inductive, and conductive coupling. In this work, only the induced AC voltage on the pipelines due to inductive coupling in steady-state conditions is studied. A holistic mathematical model for the pipelines, power lines, mitigation equipment for the induced voltage, and cathodic protection (CP) is illustrated. Potassium hydroxide polarization cells are electrically represented because these cells are considered the most common mitigation device for discharging the induced AC voltage from the pipeline to the soil. The overall model is implemented using MATLAB. The results show the profiles of induced AC voltage along the pipeline, the CP for the pipeline, the points of maximum voltage, and the influence of installing the AC mitigation units on the CP performance.

## Teaser

Chemical engineering; Electrical engineering; Electrical system planning; Electric power transmission; Power system operation; Power generation; Power system planning; AC corrosion; Induced voltage; Polarization cell; Cathodic protection.

## 1 Introduction

The effect of induced voltage produced by electromagnetic field interference from transmission lines on pipelines has been considerable interest over the past three decades. These voltages are produced due to the variation of magnetic fields produced by the currents in each transmission line's conductors with time. These voltages can be estimated using both theoretical and practical data under normal operating conditions for the overhead transmission line. Consequently, the theoretical calculation of induced AC voltage is investigated using nodal network models. The potassium hydroxide polarization cell (KOH-PC) is an electrochemical safety switch that acts as a grounding point. Therefore, the function of this polarization cell is to discharge severe induced AC voltages to ground. Then, the induced AC voltage mitigation using PCs is the most economical technique compared with different mitigation methods. Also, the induced AC voltage mitigation technique is executed based on inserting a number of KOH-PCs along the pipeline. Furthermore, the location of KOH- PCs is significant in reducing induced AC voltage along the route of the pipeline.

Moreover, the challenge of accurately predicting cathodic protection (CP) performance on the gas pipeline, especially with the presence of mitigation units due to inductive coupling, is proposed. Therefore, the construction of the polarization cell has been discussed and modeled for the first time. Finally, MATLAB software is applied to represent a model for the pipeline, induced AC voltage, mitigation units for the AC voltage, and cathodic protection system.

Nodal network analysis was applied to analyze the induced voltage in a 71.3 km long-buried gas pipeline. This pipeline was buried in parallel with a 22.9 kV power line, where the maximum induced AC voltage is 4 V [1]. In [2], the authors proposed a hybrid method based on finite element techniques to calculate both induced voltages and currents in a buried metallic pipeline, which had different configurations such as parallel and non-parallel with the power lines. This method was applied considering the coating defect and fault conditions of the power lines. The results shown that large currents may be damaged the pipeline due to the flow of the current to earth through the defects. The impact of the soil composition on the induced AC voltage along the pipeline was studied based on the finite element method. The results demonstrated that the soil constitution had a significant effect in calculating the induced AC voltage based on [3]. The induced AC voltage in a gas pipeline was calculated at different conditions using an artificial neural network. The calculation process was considered the different values of soil resistivity in addition to the normal or abnormal conditions of the neighboring power system [4]. On the other hand, a simulation software tool was developed to study the effect of high voltage (HV) power lines on a buried gas pipeline. Different models were implemented in the literature to study the effect of pipeline parameters on the calculation of induced voltage. The authors proposed that the applied simulation tool can be deal with any configuration, had a very user interface friendly so that no need to subdivide the pipelines into sections [5]. A detailed comparison between the calculations and measurements for the induced AC voltage on the metallic pipelines in case of applying different mitigation methods was introduced in [6, 7, 8]. Therefore, several studies had been carried out on the effect of electromagnetic field interference produced from the transmission line on the buried pipeline. Some of them had been focused on its effect on AC corrosion and the CP performance in the pipelines [9]. Determining the interference between OHVTLs and a metallic pipeline was significant from theoretical and experimental aspects due to the electromagnetic field effects on metallic buried gas pipelines. Additionally, different numerical models were implemented to study the inductive and conductive coupling produced due to the normal and fault conditions of the OHTL, respectively. In this study, the induced voltage was studied in various installation conditions, such as separation distance between the transmission line and the gas pipeline, and an operation state of the transmission line (normal operating condition or phase to ground fault condition). Besides, the effect of the conductor's screening factor and soil resistivity on the magnitude of the induced AC voltage along the gas pipeline was also studied. In [10, 11], the authors implemented the potential monitoring methods, such as cyclic voltammetry tests, alternating current voltammetry methods, and surface characterization to investigate the AC corrosion of cathodically protected pipeline steel. Then, the AC corrosion can be reduced by increasing the CP current to improve the CP effectiveness. Only the complete protection for the pipeline was achieved when the alternating current density reached up to 400 A/m^2^.

Moreover, in [12], the distributed source analysis concept was implemented to study the induced voltage. The electromagnetic interference problem between an existing high voltage power line and a newly designed underground pipeline with CP is studied. Then, the magnetic field on a pipeline near a high voltage power transmission line was estimated for a horizontal configuration. Additional studies had been published to investigate the calculation of induced voltages along buried gas pipelines under normal and fault conditions. Moreover, this study was also used to select a suitable location for the mitigation point for reducing induced voltages under different operating conditions, such as normal and fault conditions of the OHTL including symmetric and un-symmetric faults [13, 14].

A practical case study was performed in [15] to estimate the inductive interference caused by coupling between high voltage transmission lines and buried pipelines. Additionally, the effects of the induced AC voltage on corrosion and the CP performance of an X70 steel buried pipeline was investigated. Moreover, the effect of alternating current density on the sacrificial anode was investigated. This study was implemented to widely investigate the effect of alternating current density on the sacrificial anode with constant potential at the sacrificial anode. Also, the voltage gradient of the sacrificial anode can be affected by the electromagnetic field caused by the interfering of HVPL with the pipeline and changed its value.

In [16], the author proposed a model to determine the effect of AC voltage and current densities on the Jordanian buried gas pipeline. Additionally, defining the coating defects location along the pipeline was investigated. Furthermore, the prediction of induced voltage along the pipeline was also performed using the least-squares method. Then, the number of PCs and suitable locations for future years were examined for increasing the pipeline lifetime and decreasing the maintenance cost.

This paper introduces the modeling of metallic pipelines with interference from OHVTLs. The proposed model consists of the ICCP system and the AC voltage mitigation cell. In this study, the KOH-PC is used as the AC voltage mitigation unit, where it consists of stainless steel plates immersed in an alkaline solution (KOH) with a certain concentration to offer the immunity of the pipeline against corrosion. The KOH-PC is electrically modeled for the first time to study the most desirable mitigation conditions for the pipelines. The induced AC voltage calculations are performed before and after applying the mitigation technique to determine suitable locations for the PCs along the pipelines. Moreover, the effect of changing the number of plates in KOH-PCs on both induced AC voltages and CP performance is carried out experimentally and theoretically. There is a good agreement between the calculated results of the proposed model and the measured results in different conditions, such as with and without KOH-PCs.

## 2 Problem statement

This section introduces a comprehensive overview of the mechanisms of induced AC voltage on buried pipelines, the applied methods of the cathodic protection system for a buried pipeline, and the AC voltage mitigation cells.

### 2.1 Mechanisms of induced AC voltage

As aforementioned, three mechanisms illustrate the causes of induced AC voltage on a pipeline: capacitive, inductive, and conductive coupling. The first mechanism is that the AC voltage induced by the electric field. This mechanism does not affect pipeline in the case of fault conditions or buried pipelines under a certain level [17, 18]. The capacitive coupling, at power frequency (50 or 60 Hz), appears only on overhead pipelines. Therefore, the transversal electric field in the soil is completely negligible. The second mechanism is the inductive coupling, which can cause a severe effect on the pipeline in normal conditions. The last mechanism is the conductive coupling, in which AC voltage is induced only in case of fault to the ground. Besides, this mechanism is a rare event and lasts only for a fraction of second. On the contrary, AC corrosion has a “long-term effect”, thus the influence of conductive coupling can be ignored [19, 20]. Consequently, the inductive coupling is the only one involved in AC corrosion phenomena. Therefore, in this work, only the effect of the inductive coupling on the pipeline is studied at the normal conditions, because the pipeline is buried.

**Figure 1:**
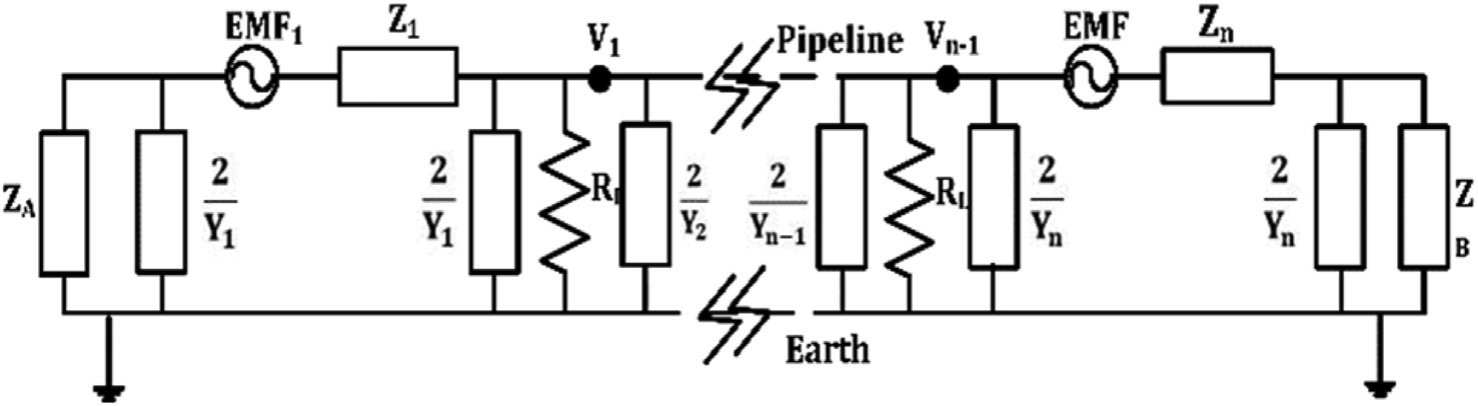
Circuit modeling of a multiple π-section pipeline.

There are various techniques to determine the induced AC voltage along the pipeline. In this study, the method tabulated in [5, 21] is followed, where high consistency between the measured and calculated results is achieved. The pipeline electrical circuit is illustrated in Figure 1, which is built based on the lossy transmission line theory. Mathematically, the pipeline can be divided into small sections; each section has uniform grounding parameters such as the soil resistance and pipeline coating resistance. In this study, the pipeline is subdivided into several sections; each section is represented as a π circuit and the length of every section equal to the tower span of the transmission line. The pipeline to soil voltage at the end of each section can then be easily calculated and measured.

From $V_{1}$ to $V_{n-1}$ represents the AC voltage at each π-section due to the magnetic field or inductive coupling for the buried pipeline, as shown in Eq. (1) [12].

(1) $$V_{n}=\frac{E_{n}}{\gamma }\left\{-\frac{Z_{A}}{Z_{A}+Z_{C}}e^{-\gamma x}+\frac{Z_{B}}{Z_{B}+Z_{C}}e^{-\gamma \left(L_{p}-x\right)}\right\}$$

where, n is an index that refers to each section for the buried pipeline, ${\text{E}}_{n}$ is the electromotive force (EMF) along the pipeline per unit length (V/km), $\;\gamma \;=\sqrt{ZY}\;$ is the pipeline propagation constant (km^−1^), $\;Z_{C}=\sqrt{Z/Y}\;$ is the lossy pipeline characteristic impedance (Ω), Z and Y are the impedance and admittance per unit length of the circuit pipeline-earth, respectively, $\;Z_{A}\;$ and $Z_{B}\;$ denote the equivalent impedance of the left side and right side of a buried pipeline, respectively (Ω), $\;L_{p}$ is the subsection length of pipeline (m) and x represents a variable distance along the buried pipeline (m).

The length induced EMF (V/Km) on the pipeline can be calculated as follows [22]:

(2) $$E_{i}=Z_{P,A}.I_{A}+Z_{P,B}.I_{B}+Z_{P,C}.I_{C}+Z_{P,E}.I_{E}$$

where, $I_{A}$, $I_{B}$, $I_{C}$, and $I_{E}$ are the phase currents in each phase conductors and ground conductors, respectively.

Additionally $\;Z_{P,A}$, $Z_{P,B}$, $\;Z_{P,C}$ and $Z_{P,E}$ are the mutual impedances between phase A, B, C, and the earth wire of the power lines, and pipeline per unit length (Ω/km), respectively.

(3) $$Z_{P,\;j}=\pi^{2}.10^{-4}.f+j4.\pi .10^{-4}.f.log_{e}\left(\frac{D_{erc}}{d_{pj}}\right)$$

where, j is an index that refers to A, B, C or E; $d_{pj}$ is the separation distance between the pipeline and line conductor j and is equal to $\sqrt{{{\left(x_{j}-x_{P}\right)}^{2}+\left(y_{j}-y_{p}\right)}^{2}}$; *f* is the system frequency and $D_{erc}$ is the depth of equivalent earth return, which equals $658.87\sqrt{\frac{\rho_{soil}}{f}}$.

The earth wire current $I_{E}$ is described in Eq. (4).

(4) $$I_{E}=\frac{-1}{Z_{EE}}\left(Z_{E,A}I_{A}+Z_{E,B}I_{B}+Z_{E,C}I_{C}\right)$$

where, $Z_{EE}$ is the self-impedance of the earth wire and is illustrated as follows:

(5) $$Z_{EE}=R_{E}+\pi^{2}.10^{-4}.f+j4.\pi .10^{-4}.f.log_{e}\left(\frac{D_{erc}}{GMR_{E}}\right)$$

where, $R_{E}$ is the footing resistance of tower, $GMR_{E}\;$ is the geometric mean radius of the ground wire and it equals to its radius $K_{sf}r_{E}$ , and $\sqrt{d_{E}.r_{E}.e^{-0.25}}$ for one and two earth wires, respectively. $K_{sf}\;$ is the conductor's stranding factor provided by the conductor manufacturer, $d_{E}$ is the distance between two ground wires, and $r_{E}$ is the radius of ground wire. Moreover, $Z_{E,A}$, $Z_{E,B}$ and $Z_{E,C}$ are the mutual impedances between the earth wire and the phases of the power system per unit length, which can be expressed by Eq. (6).

(6) $$Z_{E,j}=0.04935+j0.14468.log_{\text{e}}\left(\frac{D_{erc}}{D_{E,i}}\right)$$

where, j is an index that refers to A, B, C; $D_{Ej}$ is the separation distance between the pipeline and line conductor j , and is equal to $\sqrt{{{\left(x_{j}-x_{E}\right)}^{2}+\left(y_{j}-y_{E}\right)}^{2}}$.

While the series impedance of a buried pipeline with earth return per unit length (Ω/Km) is given by [5, 22]:

(7) $$Z_{n}=\frac{\sqrt{\rho_{p}\mu_{p}f}}{3.163r_{p}}+\pi^{2}.10^{-4}.f+j\left[\frac{\sqrt{\rho_{p}\mu_{p}f}}{3.163r_{p}}+4.\pi .10^{-4}.f.ln\left(\frac{D_{erc}}{r_{p}}\right)\right]$$

where, $\rho_{p}$ is the pipeline's resistivity, $\mu_{\text{p}}$ is the relative permeability of the pipeline's metal (typically about 300 for steel), f is the system frequency, and $r_{p}$ is the pipeline's outer radius.

For pipelines with high resistivity coated materials, the equivalent shunt admittance Y consists of the coating's admittance $Y_{c}$ in series with the external earth admittance $\;Y_{e}$.$\;Y_{e}$ should be added in the total pipeline admittance calculation due to earth and that, in case of good coatings, it can be neglected. Therefore, the shunt admittance of a buried pipeline is given by:

(8) $$Y_{n}=Y_{c}=\frac{2000.\pi .r_{p}}{\rho_{c}.\delta_{c}}+j\frac{\pi .r_{p}.f.\varepsilon_{c}.10^{-6}}{9.\delta_{c}}$$

where, $Y_{c}\;$ is the coating's admittance per unit length (℧/km), $\;\rho_{c}$ is the pipeline coating's electrical resistivity (Ω.m), ${\text{δ}}_{\text{c}}$ is the coating thickness (m), and ${\text{ε}}_{\text{c}}$ is the coating's relative permittivity.

### 2.2 Cathodic protection principle

CP is an effective method often used to prevent the corrosion of metallic pipelines. A coating process is also an effective method for protecting a pipeline against corrosion; however, this method does not provide complete protection for the pipeline. Therefore, the coating process must be associated with connecting the pipeline to an element that is more active than the pipeline. To achieve complete cathodic protection for the pipeline, it is extensively either subjected to an impressed current system or attached to a sacrificial anode (e.g. aluminum, zinc, or magnesium). Therefore, there are two general methods of cathodic protection systems, namely impressed current (ICCP) and sacrificial anode (SACP). Owing to the effect of the electromagnetic field on the sacrificial anode, it is essential to develop methods to eliminate this problem. One of these methods is the impressed current cathodic protection (ICCP), which uses as a means to protect the pipeline against AC corrosion. In this method, the output current of CP is injected in the pipeline by a rectifier.

**Figure 2:**
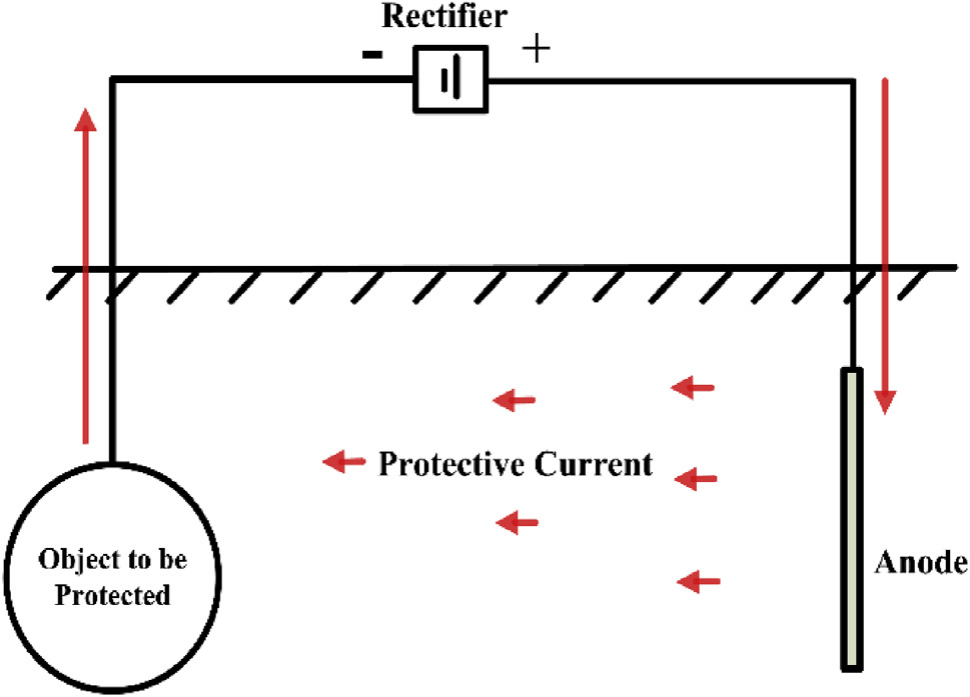
The model of impressed current cathodic protection (ICCP) system.

Figure 2 shows the ICCP system, an electric direct current is injected along the pipeline from an external DC supply (usually a rectifier). This source is electrically connected between an anode and the pipeline (cathode). This type of anode is usually known as a silicon-iron anode, and it is an alloy of silicon, magnesium, carbon, iron and, sometimes, chromium. According to ASTM A 518-80, this material can give a current density of approximately 10–30 A/m^2^ in soil or freshwater and 10–50 A/m^2^ in seawater [23, 24, 25, 26, 27]. In the second method, the SACP system, the pipeline is electrically connected to the groups of sacrificial anodes. The usual rule is to preserve the pipeline at a constant potential between -0.85 and -1.3 V (with respect to a copper electrode/saturated copper sulfate solution Cu/CuSo_4_) [16,28,29].

The cathodic protection criterion for buried pipelines is based on AC and DC density. Consequently, it is necessary to study the effect of different CP levels on AC corrosion. AC corrosion could be mitigated to a negligible corrosion rate if AC density is below 100 A/m^2^. Additionally, DC densities lower than 0.1 A/m^2^ cause corrosion due to the lack of CP and DC densities greater than 20 A/m^2^ cause disbonding between the metal and coating of the pipelines. This leads to a galvanic cell, and the probability of pipeline corrosion is increased. Therefore, the CP current densities should be kept in the 1–20 A/m^2^ range to keep the pipeline in the immunity zone [21].

The major merits of ICCP systems over SACP systems are that the driving potential of an ICCP system is not reduced by the corrosion potential of an active metal. Additionally, ICCP has more flexibility in selecting the suitable driving potential and adjusting it after installation of the CP system. Then, ICCP may also give additional flexibility in selecting the location of the anode beds to achieve an optimum distribution of protective current along the pipeline with a minimum of AC interference. Therefore, the ICCP system has been applied with the long length pipelines [24].

As described previously, the drawn current from the DC source to the pipeline (rectifier) depends on the soil conductivity, and the change of the DC applied voltage, which can be described by Eq. (9) [30]:

(9) $$i_{l}=-\sigma_{l}.\nabla \emptyset_{l}$$

where, $i_{l}$ is the electrolyte (soil) current density vector, $\sigma_{l}$ is the soil conductivity, and $\emptyset_{l}$ is the DC voltage to anode bed potential. The default insulation condition for all boundaries of the buried pipeline is described as follows:

(10) $$\nabla .i_{l}=0\;$$

**Figure 3:**
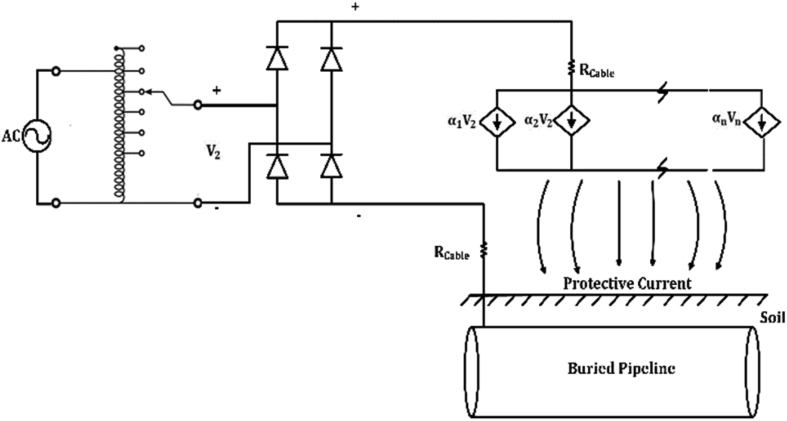
Equivalent circuit of cathodically protected pipeline.

Figure 3 shows a detailed schematic for the ICCP system, it should be noted that the negative point is connected to the pipeline to keep the metal highly negative, and the positive point is connected to the silicon-iron anodes. Each silicon-iron anode is a source of Direct current, but this source depends on the DC input voltage. It could be assumed that all anodes have the same dependent current source, all sources are connected in parallel, and the total current is $\alpha V_{DC}$.

The impressed current cathodic protection (ICCP) system is designed to eliminate the telluric voltage fluctuations, which are produced near the transformer-rectifier location. Therefore, the total current output (I_o_) will be the sum of the cathodic protection current (I_cp_) and the telluric current (I_t_), which can be described as follows:

(11) $$I_{o}=I_{cp}\;+\;I_{t}$$

To mitigate the telluric current, impressed current cathodic systems should not be operated in constant current mode. This mode may reduce the cathodic protection current by the amount of the telluric current through the rectifier. Therefore, the voltage and current output of the transformer-rectifier should be changed automatically in response to the measured pipeline to soil potential [31].

### 2.3 AC voltage mitigation units

The process of mitigating the induced AC voltage along the route of the pipeline is usually represented as the most important criterion in preventing metal corrosion at coating impurity locations, as well as reducing the exposure of personnel to electrical hazards. Different techniques can be implemented to mitigate induced AC voltages along the gas pipelines, such as cancellation wires, gradient control wires, insulating joints, and PCs. In this research, the process of mitigating the induced AC voltage is carried out by connecting KOH-PCs to the pipeline. This mitigation technique is evaluated theoretically and experimentally.

#### 2.3.1 Polarization cell

**Figure 4:**
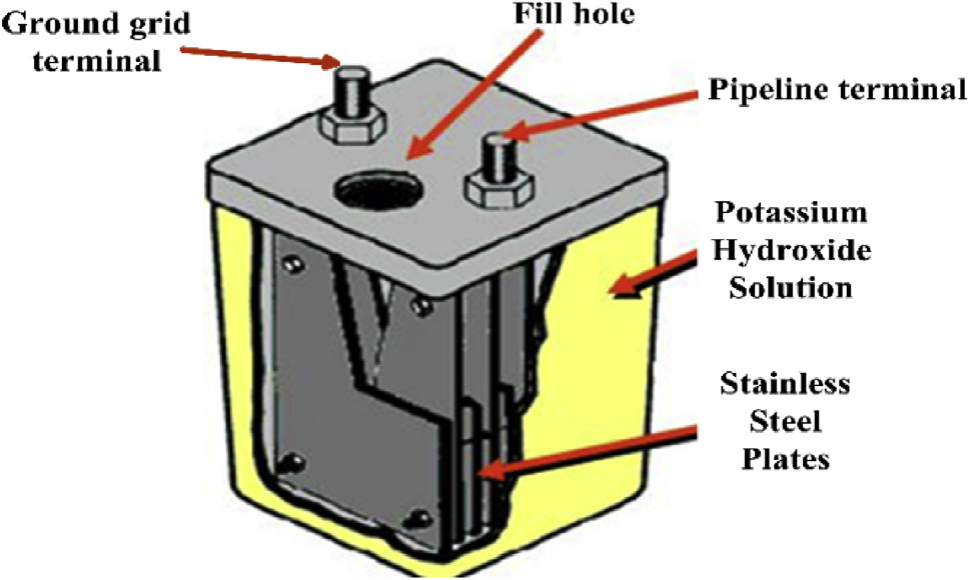
KOH-polarization cell construction.

Initially, the KOH-PC is an electrochemical safety switch that acts as a grounding path. Therefore, the function of this polarization cell is to dissipate severe induced AC voltages to ground. This means that it mainly has two terminals; one is connected to the pipeline and the other terminal is connected to the earth. For discrimination, the pipeline terminal is termed as the upper terminal and the earth terminal is known as the lower terminal. However, the cell will allow alternating current to dissipate quickly while blocking direct current to improve the CP performance. A KOH-PC consists of multiple pairs of stainless-steel plates that are immersed in a 30 wt. % KOH electrolyte solution, as shown in Figure 4. This concentration provides corrosion immunity for iron [32, 33, 34].

To obtain accurate results, the KOH-PC is modeled based on the electrical circuit representation of all its parts. This cell mainly contains two terminals, each terminal has a group of stainless-steel plates, and these plates are arranged in a nested way. In other words, there are separation distances between the plates, and each upper terminal plate is followed by a lower terminal plate and so on. If counting starts from the first upper terminal, it will be finished at the last lower terminal plate. This means, the number of upper terminal plates equals the lower terminal plate's number, and they are separated by equal separation distances.

For a certain level, all these plates are submerged in a 30 wt. % KOH solution. The first plate is connected to the pipeline and has the pipeline voltage. Besides, the second plate two is connected to the earth and has approximately a zero voltage. Additionally, the two plates are separated from each other by a certain distance, $\;d_{12}$. This distance is occupied by two materials, air and the KOH solution. Consequently, two capacitors result between the 1^st^ and 2^nd^ plate, one $\left({\text{C}}_{{\text{air}}_{12}}\right)$ due to the air and the other $\left(C_{KOH_{12}}\right)$ due to the KOH solution. The capacitance due to air is negligible [34, 35].

(12) $$C_{air_{\text{ij}}}=\frac{\varepsilon_{0}.A_{air}}{d_{\text{ij}}}$$

(13) $$C_{KOH_{\text{ij}}}=\frac{\varepsilon_{0}.\varepsilon_{r}.A_{KOH}}{d_{\text{ij}}}$$

This capacitance is connected in parallel with a resistance that represents the KOH path from plate one to plate two, where the length of this path is the separation distance between the two plates. The cross-sectional area of this path is the area of the submerged part in the KOH solution. Consequently, the resistance of the path is calculated by Eq. (14).

(14) $$R_{KOHij}=\frac{d_{\text{ij}}}{\sigma_{KOH}.A_{pij}}$$

where, i and j are indexes that refer to the number of plate,  i≠j, $\sigma_{KOH}$ is the conductivity of this solution and it is calculated by Eq. (16), and $A_{pij}$ is the area of the submerged part in the solution.

**Figure 5:**
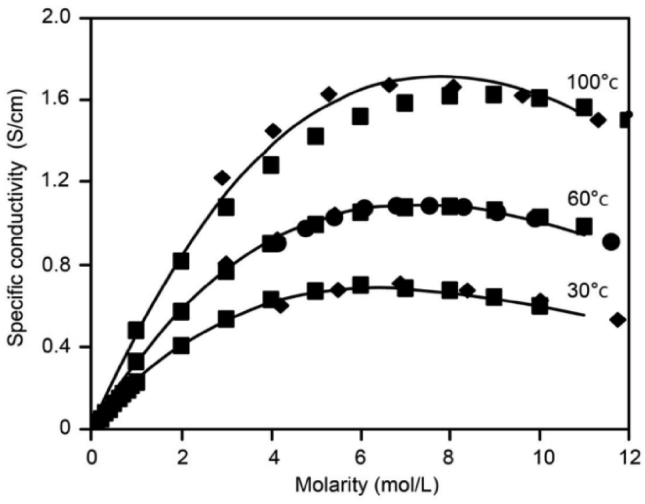
Specific conductivity vs. molarity for different temperatures.

Measurements are extensively tested for a 27 wt.% KOH solution at a wide range of temperatures, from 0 to 75 °C, using a modified Jones conductivity cell. This cell is constructed from polymethylmethacrylate blocks to avoid any attack caused by any caustic electrolyte. This cell consists of a cylindrical cavity with a 1.1 cm diameter, and two platinized-platinum parallel electrodes. A Radiometer CDM83 is implemented at 50 kHz to measure the KOH conductivity. Two commonly concentration units are applied in recording the test data, such as the concentration of KOH (wt.% KOH) and molarity [36, 37, 38]. Eq. (16) is applied to convert between these units;

(15) $$M=\frac{wt\text{\%}\left(KOH\right).D_{KOH}}{100.M_{\text{W}}}$$

where, $D_{KOH}$ is the density of the KOH solution in $kg/m^{3}$, which equals $G.e^{\left(0.0086.wt\text{\%}\right)}$ , where G is a constant related to the test temperature, and $M_{\text{W}}$ is the molar mass of KOH in g/mol. Finally, an empirical formula for specific conductivity related to the concentration of KOH in molarity, and temperature in Kelvin is represented by Eq. (16):

(16) $$\sigma_{KOH}=k_{1}.M+k_{2}.M^{2}+k_{3}.M.T+k_{4}.\frac{M}{T}+k_{5}.M^{3}+k_{6}.M^{2}.T^{2}$$

where, ${\text{σ}}_{\text{KOH}}$ is the specific conductivity in S/cm, M is the molarity in mol/L , and T is the temperature in Kelvin. The values of $\;k_{1}$, $k_{2}$, $k_{3}$, $k_{4}$, $k_{5}$ and $k_{6}$ are −2.041, −0.0028, 0.005332, 207.2, 0.001043, and −0.0000003, respectively. Additionally, this equation is represented by Figure 5.

Each plate is represented by a resistor $R_{P_{i}}$; the odd-numbered resistors are the plate resistors connected to the pipeline, while the even resistors are the plates connected to the earth terminal. The value of these resistances are determined by Eq. (17):

(17) $$R_{P_{i}}=\frac{\rho_{s.s}.L_{P_{i}}}{A_{P_{i}}}$$

where, $\rho_{s.s}$ is the resistivity of the stainless-steel, $L_{P_{i}}$ is the length of plate i, and $A_{P_{i}}$ is the cross-sectional area for the same plate.

**Figure 6:**
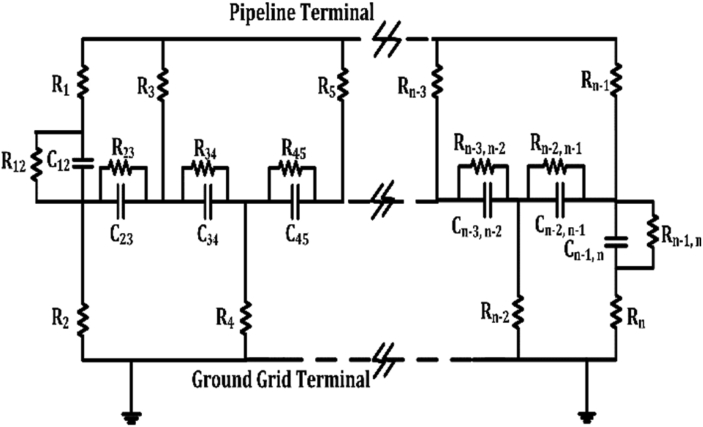
Electrical circuit model of KOH-Polarization cell.

Finally, the equivalent circuit of the KOH-PC is illustrated in Figure 6. The electrical model for the pipeline, ICCP system, and AC mitigation unit is introduced in this section. Therefore, this model is modeled using MATLAB to carry out the analysis of induced AC voltage, and DC CP voltage along the pipeline, in addition to the simulation results are compared to real measurements from the field. Therefore, all KOH components, such as plates and solution, should be taken into account. The following section shows the main results of induced AC voltage before and after mitigation, in addition to the impact of mitigating the induced AC voltage on the performance of the CP. Moreover, the effect of changing the number of plates in the KOH-PCs on the induced AC voltage, and cathodic protection DC voltage is shown.

**Figure 7:**
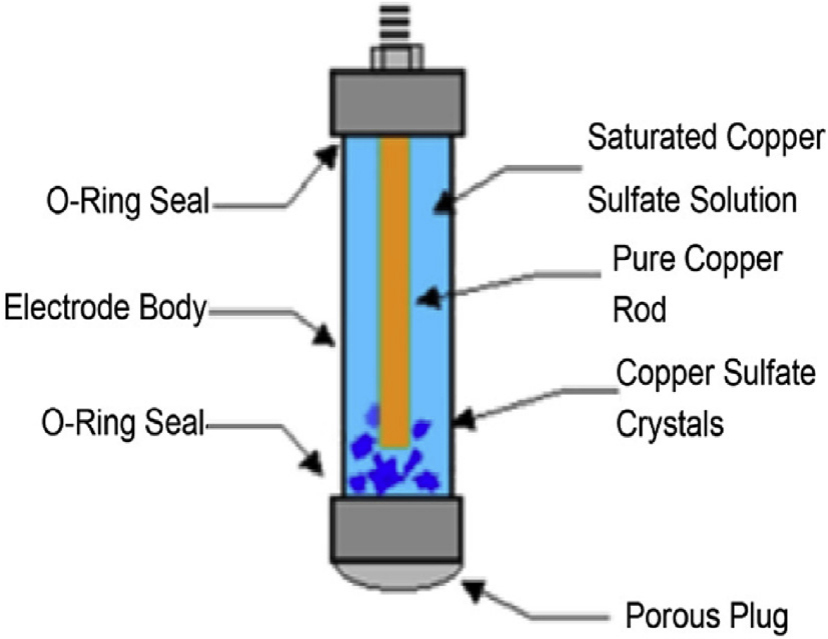
The Cu–CuSO_4_ reference electrode construction.

**Figure 8:**
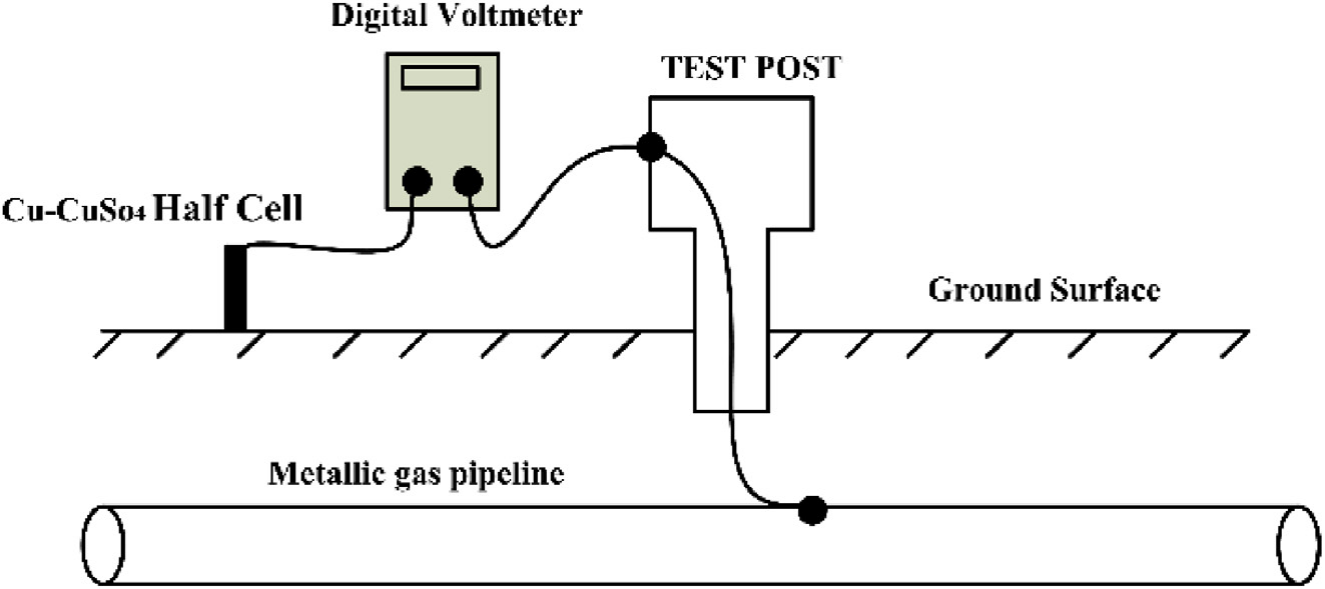
Measurements of the pipeline DC and AC voltages.

In this study, the measurements of induced AC voltage and DC voltage CP along the 72 km gas pipeline are experimentally performed. These measurements are carried out using a digital ac voltmeter, which is connected between the pipeline test point and a portable reference electrode. This electrode is known as a Cu–CuSO_4_ reference electrode, which is considered as a simple electrochemical cell that uses a chemical reaction to generate an electric current. One common type of half-cell includes a copper electrode immersed in copper sulfate electrolyte, which is illustrated in Figure 7 [21]. The following measurement technique is presented in Figure 8. Measurement of induced AC voltage (AC pipeline to soil potential) and DC potential is simultaneously performed by connecting the one terminal of this voltmeter to the reference electrode (Cu-CuSo_4_), and the other is connected to the test port at the pipeline, as shown in Figure 8. For the AC voltage measurement, the voltmeter is set on the AC mode, while it is in DC mode for the DC voltage measurement. To enhance the effectiveness of voltmeter reading, the electrode should be impressed in wet soil with freshwater to give a good connection. Moreover, the reference electrode used in the measurement should be located perpendicular to the interfering overhead transmission line to avoid any noise voltage induced on it. The measurement of induced AC voltage and DC voltage cathodic protection along the pipeline is carried out for several days.

## 3 Application to case study

**Figure 9:**
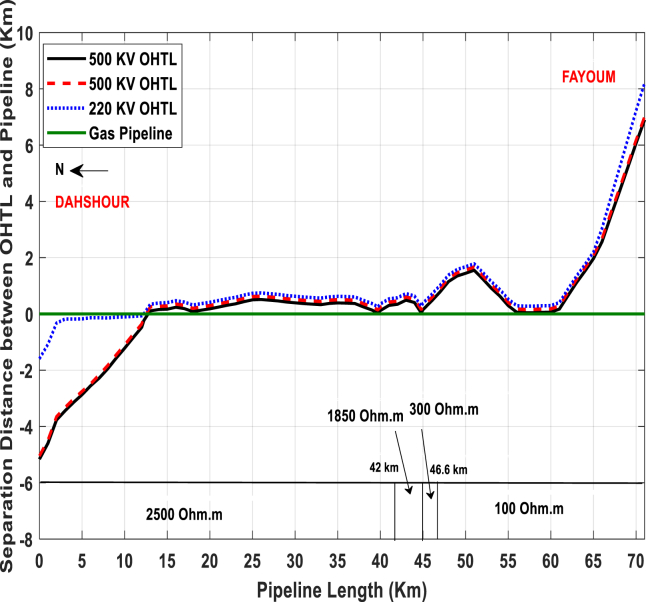
Fayoum gas co. pipeline-power line separation distances.

The studied pipeline is a natural gas metallic pipeline owned by Fayoum Gas Company, Egypt. This pipeline has been used in many dissertations and research articles since 2013 [12,18], and complementary studies are applied in this work for the same pipeline. As is known, this line is 72 km long and is a neighbor to three overhead high voltage transmission lines (El-Kurimate-Cairo power line, Samaloute-Cairo power line, and Dimo-6^th^ of October power line), as shown in Figure 9. Besides, the pipeline has a diameter of 16 inches or 0.4064 m. It is plated by three layers of High-Density Polyethylene (HDPE), which has a coating resistance of 10^6^ Ω/m^2^, the relative permittivity of 5, and its thickness is 4 mm. It is buried at a depth of 1.5 m with the soil resistivity changes from 2500 to 100 Ω m through the pipeline route.

**Fig. 10:**
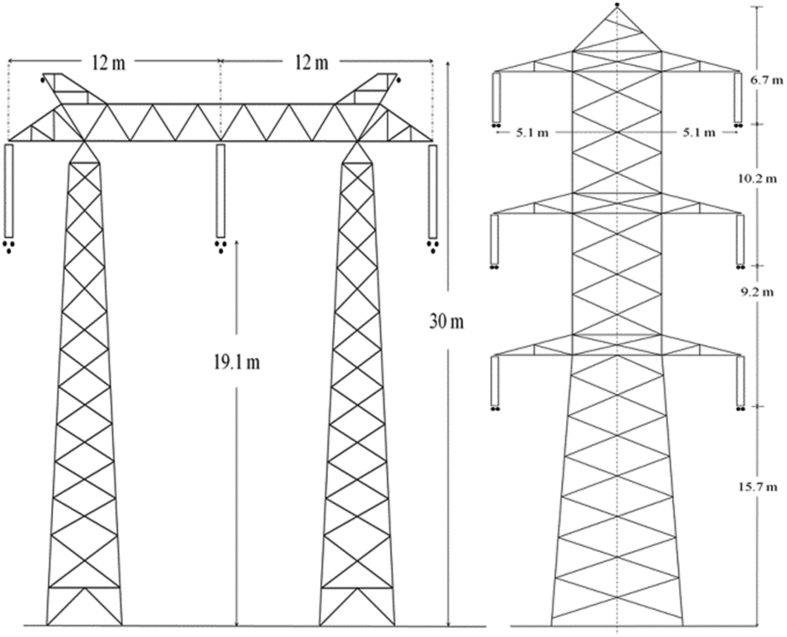
The towers for the overhead high voltage transmission lines.

Two OHVTLs have a voltage of 500 kV, and the tower for these lines carries only one three-phase power circuit and two earth wires. The last OHVTL has a voltage of 220 kV and carries two three-phase power circuits and one earth wire. Figure 10 and Table 1 show the power line data and tower dimensions. The left image in Figure 10 shows the 500 kV OHVTL towers, and the right one shows the 220 kV OHVTL tower.

**Table 1:** Technical data on Power lines.

Parameter	Value	
Rated Power in MVA	575	158
Line to line voltage in kV	500	220
Power lines length in km	124	90
No. of tower circuits	1	2
No. of phase conductors	3	2
Conductor diameter in mm	30.6	27
The conductors spacing in cm	47	30
Towers span in meter	400	360
Vertical height of conductor at tower in meter	19.1	15.7
No. of overhead earth wires	2	1
The height of overhead earth wire in meter	30	41.8
Diameter of overhead earth wire in mm	11.2	13.6

**Fig. 11:**
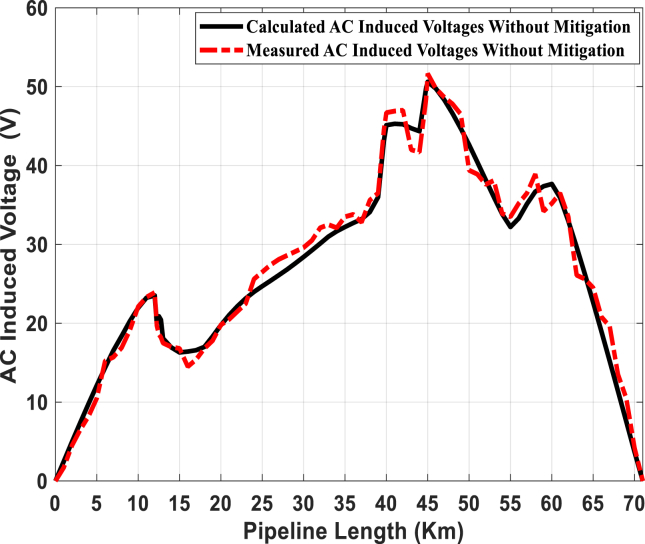
Comparison between measured and computed induced AC voltage along the pipeline.

Both the electrical circuit model of the pipeline and the KOH-polarization cell are validated by comparing the measured and computed results for the Fayoum gas metallic pipeline. Figure 11 illustrates the measured and calculated induced AC voltage on the pipeline by the inductive coupling at steady-state conditions, where Eq. (1) is applied to determine this voltage. From Figures 9 and 11, it is easy to note that the points of the lowest separation distance between the power lines and pipeline have the highest value of induced AC voltage. The lowest separation distance points are allocated at 12.75, 18, 39.7, 44.8 and 58 km from the start of the pipeline, where the values of the induced AC voltage are 20.43, 17, 43.12, 49.79 and 35.93 V, respectively.

The calculated induced AC voltage is in a good agreement with the measured voltage. However, there are some small variations between the calculated and measured induced AC voltage. These are due to the complexity in recording the values for the line current of transmission lines at the instant of the pipeline to soil potential measurements. Besides, the soil resistivity that may change from point-to-point; it may be homogeneous and non-homogeneous at some points based on the weather conditions. Moreover, the time between the measurement of the soil resistivity and induced AC voltage along the pipeline is a long period. In this study, some simplifying assumptions have been considered, such as the homogeneous surrounding soil.

**Fig. 12:**
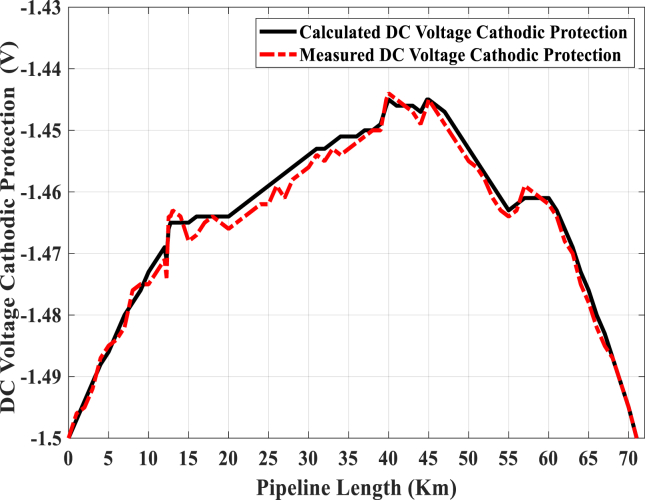
CP DC voltage of the pipeline under the normal condition without mitigation.

There are two impressed current cathodic protection (ICCP) stations for this pipeline, one at the start point and the other is at the end of the pipeline. Additionally, the CP is measured in negative DC volts. Therefore, the CP values are high at both the start and end of the pipeline, and it reduces to the lowest value in the middle of the pipeline. From Figure 12, the CP potential varies between -1.5 and -1.44 $V_{DC}$, where this value is about -1.5 $V_{DC}$ at the pipeline line terminals and -1.44 $V_{DC}$ in the middle of the pipeline. The effective value of DC potential recommended for completely cathodic protection of steel pipeline buried in the soil must be ranged between -0.85 to -1.5 $V_{DC}$ to achieve complete protection against DC corrosion, which originates from direct stray current activities [18].

**Fig. 13:**
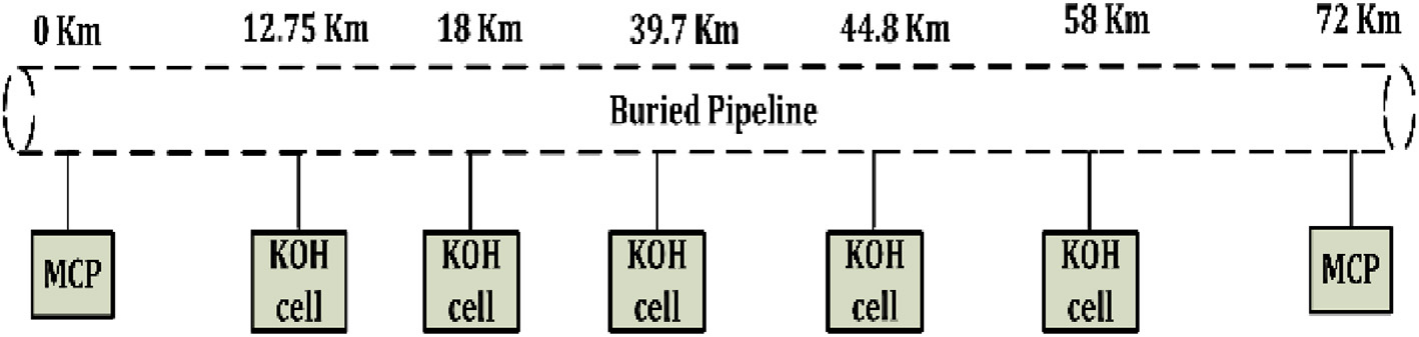
The complete configuration for the installed KOH-PCs and the main ICCP units for the pipeline.

KOH-PCs are installed at the points of the highest values of induced AC voltage to eliminate any electrical hazards that could occur due to this voltage. These hazards may cause an electrical shock for the operators or may cause the pit corrosion for the pipeline. The pit corrosion usually accelerates the corrosion of the pipeline's body and causes the damage of its coating. Therefore, it is necessary to design the mitigation system to enhance the complete protection for the pipeline. Figure 13 shows a complete configuration for the installed KOH-PCs and the main ICCP units for the pipeline.

The high current density flows to the soil due to inducing a high AC voltage when the coating has a defect, and AC corrosion occurred at a high rate. Here, the KOH-PCs play a key role, where the high induced AC voltage discharges into the soil in a safe way to avoid any hazard for the human or the AC corrosion occurrence. Accordingly, the computed results of the induced AC voltages along the length of the pipeline show that many points have a high AC voltage. Therefore, the KOH-PCs should be installed at the high voltage points to maximize the discharging of alternating current from the pipeline to the soil. The proposed study is based on two main models of KOH-PCs; the first model has 25 stainless steel nested plates (PC-25), and the last has 50 plates (PC-50). Moreover, the challenge of accurately predicting cathodic protection (CP) performance on the gas pipeline, especially with the presence of mitigation units due to the inductive coupling, is proposed.

**Fig. 14:**
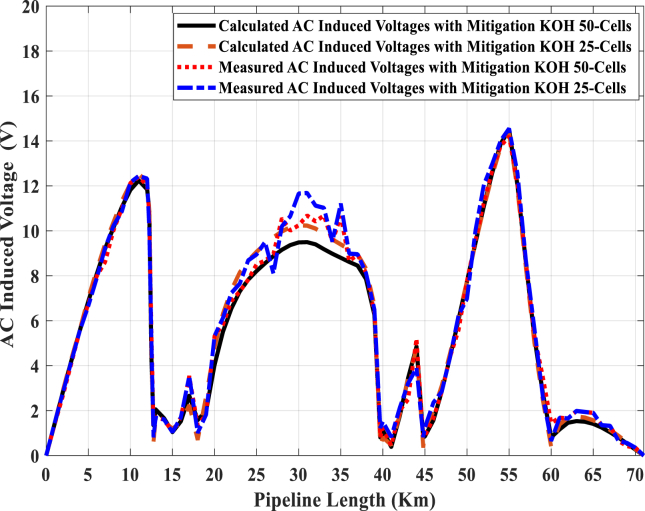
Measured and calculated pipeline induced AC voltage.

Experimentally, it is noted that the discharging of induced AC voltage will be increased when the KOH-PC is installed at a high voltage point. Consequently, the number of plates in the KOH-PCs are increased, the discharged current is increased. Finally, Figure 14 illustrates a comprehensive comparison between the measured and calculated induced AC voltage at the pipeline using the PC-25 and PC-50. These cells are installed at 12.75, 18, 39.7, 44.8 and 58 km from the start of the pipeline, and the data for these points are summarized in Table 2. The maximum value of induced AC voltage along the pipeline after installing KOH-PCs does not exceed 15 V (RMS), according to NACE International Recommended Practice RP0177 [39].

**Table 2:** Comparisons for the highest induced AC voltage points using different KOH-PCs.

Point (km)		12.75	18.00	39.70	44.80	58.00
Without PC	Calculated	20.43	17	43.12	49.79	36.69
	Measured	18.2	16.8	44.5	48.4	38.9
PC-25	Calculated	1.353	1.582	0.81	0.803	5.293
	Measured	1.368	1.352	0.823	0.812	5.352
PC-50	Calculated	0.611	0.6992	0.379	0.353	5.029
	Measured	0.810	1.012	1.231	0.821	5.18

Therefore, the KOH-50 cell gives the best performance in the discharging of the induced AC voltage, where the electrical paths from the pipeline to the soil are increased, which lead to significantly suppress the induced AC voltage. It is noticed that there are some points; the satisfactory agreement between measured and calculated results is achieved in the case of using the PC-25 and PC-50. Moreover, the quite difference is due to the complexity in recording the values of line current and soil resistivity at the instant of induced AC voltage measurement. Also, the line current and soil resistivity change from season to season, so the induced AC voltage is measured in different seasons.

Besides, from Table 2, the effect of the KOH-25 cell on the discharging of AC voltage is good, but at the same time, the differences between the calculated and measured results are very small compared to the KOH-50 cell. However, it can be noted that the induced AC voltage will be reduced, if the KOH-PC is injected on the pipeline, in addition to the DC voltage of cathodic protection may be significantly affected. On the other hand, the KOH-PC introduces an easier bridge to allow the discharging of direct current to the soil. A leakage direct current discharges to the soil through the electrical path, that represented by the KOH-PC. Consequently, the electrical paths, from the pipeline to the soil, are increased by increasing the number of plates in KOH- PC, so the discharge direct current to the soil is incrementally increased using a KOH-50 cell rather than a KOH-25 cell. Therefore, the DC voltage of cathodic protection is significantly reduced at some points, especially by installing the KOH-50 cell.

**Fig. 15:**
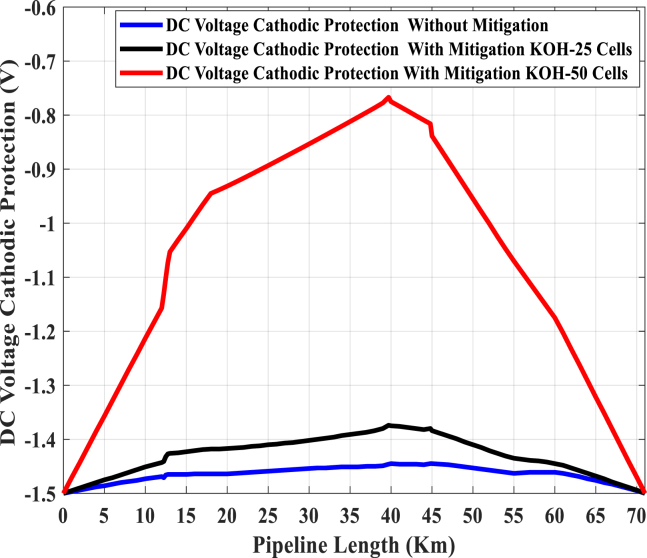
DC voltage cathodic protection for different conditions.

Figure 15 illustrates the DC voltage of CP in three different cases. The blue line shows the CP voltage performance before installing the KOH-PCs. The black line illustrates this performance after installing the KOH-25 cells, and the red line describes the CP performance after installing the KOH-50 cells. As noted in Figures 14 and 15, the KOH-50 cell gives the best performance for the discharging of the induced AC voltage, but its CP performance is very risky. The risky situation comes from the values of CP voltage at some points being near the minimum allowable limit of CP voltage (-0.85 $V_{DC}$) defined by the NACE standard. From the two figures, the influence of the KOH-25 cell on the discharging of the induced AC voltage is good, but at the same time, it does not draw a lot of direct currents.

## 4 Conclusion

In this paper, a holistic model for the pipeline is presented, where this model considers the coupling between the pipeline and neighboring OHVTLS. This model is linked with the ICCP system model. Moreover, the performance of induced AC voltage and CP voltage without mitigation is studied only in steady-state conditions. The points of highly induced AC voltage are selected to install the KOH-PCs, which minimize the hazards of high voltage on the pipeline. The electrical model for the KOH-PC is completely described to study the discharging process and the influence of these cells on both induced AC and CP voltage. Therefore, two models of KOH-PC are presented in this study. Consequently, it is necessary to check the cathodic protection level along the pipeline route in an adequate limit after the inserting of polarization cells. It is noticed that the effect of changing the number of plates in the KOH-PCs has a significant impact on both induced AC and DC voltage, due to the variation of the KOH-PCs’ equivalent impedance. Therefore, if the number of plates in the KOH-PCs is increased, the discharging of both AC and DC voltage will be increased. As a result, the proposed KOH–PC model shows more precise performance compared to other mitigation methods. It is noted that the proposed KOH–PC model can be used for the real-time discharging the induced AC voltage with a slightly negative impact on the negative DC voltage.

## Declarations

### Author contribution statement

Mostafa A. Al-Gabalawy & Mohamed A. Mostafa: Conceived and designed the experiments; Performed the experiments; Analyzed and interpreted the data; Contributed reagents, materials, analysis tools or data; Wrote the paper.

Abdel Salam Hamza: Conceived and designed the experiments; Analyzed and interpreted the data; Contributed reagents, materials, analysis tools or data; Wrote the paper.

Shimaa A. Hussien: Contributed reagents, materials, analysis tools or data; Wrote the paper.

### Funding statement

This research was funded by the Deanship of Scientific Research at Princess Nourah bint Abdulrahman University through the Fast-track Research Funding Program.

### Competing interest statement

The authors declare no conflict of interest.

### Additional information

No additional information is available for this paper.

## References

[1] Lee H., Ha T., Ha Y., Bae J., Kim D. (2004). “Analysis of voltages induced by distribution lines on gas pipelines. International conference on power system technology, Powercon.

[2] Christoforidis G., Labridis D., Dokopoulos P. (2005). A hybrid method for calculating the inductive interference caused by faulted power lines to nearby buried pipelines. IEEE Trans. Power Deliv..

[3] Christoforidis G., Labridis D., Dokopoulos P. (2005). Inductive interference on pipelines buried in multilayer soil due to magnetic fields from nearby faulted power lines. IEEE Trans. Electromagn C..

[4] Al-Alawi S., Ellithy K., Al-Badi Abd. (2005). An artificial neural network model for predicting gas pipeline induced voltage caused by power lines under fault conditions. COMPEL.

[5] Bortels L., Deconinck J., Munteanu C., Topa V. (2006). A general applicable model for AC predictive and mitigation techniques for pipeline networks influenced by HV power lines. IEEE Trans. Power Deliv..

[6] Ismail I H. (2007). Effect of oil pipelines existing in an HVTL corridor on the electric-field distribution. IEEE Trans. Power Deliv..

[7] Kopsidas K., Cotton I. (2008). Induced voltages on long aerial and buried pipelines due to transmission line transients. IEEE Trans. Power Deliv..

[8] Southey R.D., Dawalibi F.P., Vukonich W. (1994). Recent advances in the mitigation of voltage of AC voltages occurring in pipeline located closed to electric transmission lines. IEEE Trans. Power Deliv..

[9] Şteţ D., Micu D., Czumbil L., Darabant L., Ceclan A. (2012). Simulation of interferences between power lines and gas pipelines in unbalanced phase currents state. COMPEL.

[10] Fu A., Cheng Y. (2012). Effect of alternating current on corrosion and effectiveness of cathodic protection of pipelines. Can. Metall. Q..

[11] Xu L., Su X., Cheng Y. (2013). Effect of alternating current on cathodic protection on pipelines. Corrosion Sci..

[12] Gouda O., Zain A., Al-Gabalawy M. (2013). Effect of Electromagnetic field of overhead transmission lines on the metallic gas pipelines. Electr. Power Syst..

[13] M'hamed O., Mourad Z. (2014). AC corrosion induced by high voltage power line on cathodically protected pipeline. International conference on control, engineering & information technology (CEIT’14).

[14] Kamal N., Zain A., magdy M. (2015). Mitigation of induced voltages and AC corrosion effects on buried gas pipeline near to OHTL under normal and fault conditions. Electr. Power Syst. Res..

[15] Ouadah M., Touhami O. (2017). Method for diagnosis of the effect of AC on the X-70 pipeline due to an inductive coupling caused by HVPL. IET Sci. Meas. Technol..

[16] Kamar A. (2018). Location estimation of coating defects and mitigation of induced AC voltages along buried gas pipeline. IET Sci. Meas. Technol..

[17] Radwan R., Amer R., Emam A. (2002). Combined electric and magnetic field on metallic pipelines near HV transmission lines. Cigre session.

[18] Al-Gabalawy M. (2011). Electrical Hazards on Natural Gas Metallic Pipelines Due to High Voltage Overhead Transmission Lines.

[19] (2000). AS/NZS 4853: ' Electric Hazards on Metallic Pipelines.

[20] Gupta A. (2008). A Study on High Voltage AC Power Transmission Line Electric and Magnetic Field Coupling with Nearby Metallic Pipelines.

[21] Zhang H., Karady G., Hunt J. (May 2011). Effect of various parameters on the inductive induced voltage and current on pipelines. IEEE Trans. Power Deliv. Ariz. State Univ..

[22] Tleis N. (2008). Power Systems Modelling and Fault Analysis Theory and Practice.

[23] (1995). Guide on the Influence of High Voltage AC Power Systems on Metallic Pipelines.

[24] Laoun B., Niboucha K., Serir L. (2008). Cathodic protection of a buried pipeline by solar energy. Revue des Energies Renouvelables.

[25] ISO 12473 (2017). General Principles of Cathodic Protection in Seawater.

[26] DS 91 (2017). Standard for the Selection and Design of Cathodic Protection.

[27] ASTM A518/A518M – 99 (2018). Standard Specification for Corrosion-Resistant High-Silicon Iron Castings.

[28] NACE Standard RP0169 (2007). Control of External Corrosion on Underground or Submerged Metallic Piping Systems.

[29] ISO 15589-1, “Petroleum and Natural Gas Industries—Cathodic Protection of Pipeline Transportation Systems; Part 1 On-Land Pipelines”.

[30] Qiao G., Guo B., Ou J., Xu F., Li Z. (2016). Numerical optimization of an impressed current cathodic protection system for reinforced concrete structures. Construct. Build. Mater..

[31] NACE International, 2005 (July 2008). Cp 3–Cathodic Protection Technologist Course Manual.

[32] BS EN 15280 (2013). Evaluation of AC Corrosion Likelihood of Buried Pipelines Applicable to Cathodically Protected Pipelines.

[33] NACE, SP21424 (2018). Alternating Current Corrosion on Cathodically Protected Pipelines: Risk Assessment, Mitigation, and Monitoring.

[34] Revie R.W. (2019). Oil and Gas Pipelines - Integrity and Safety Handbook.

[35] Naidu M., Kamaraju V. (2004). High Voltage Engineering.

[36] Gilliama R., Graydonb J., Kirkb D., Thorpe S. (2007). A review of specific conductivities of potassium hydroxide solutions for various concentrations and temperatures. Int. J. Hydrogen Energy.

[37] See D., White R. (1997). Temperature and concentration dependence of the specific conductivity of concentrated solutions in potassium hydroxide. J. Chem. Eng. Data.

[38] Allebrod F., Mollerup P., Mogensen M. (2012). “Electrical conductivity measurements of aqueous and immobilized potassium hydroxide. Int. J. Hydrogen Energy.

[39] NACE Standards (2000). Mitigation of Alternating Current and Lightning Effects on Metallic Structures and Corrosion Control Systems.

